# Modulation of Liver Inflammation and Fibrosis by Interleukin-37

**DOI:** 10.3389/fimmu.2021.603649

**Published:** 2021-03-04

**Authors:** Steffeni Mountford, Maria Effenberger, Heidi Noll-Puchta, Lucas Griessmair, Andrea Ringleb, Sonja Haas, Gerald Denk, Florian P. Reiter, Doris Mayr, Charles A. Dinarello, Herbert Tilg, Philip Bufler

**Affiliations:** ^1^Department of Pediatrics, Dr. von Hauner Children's Hospital, Ludwig-Maximilians-University Munich, Munich, Germany; ^2^Department of Internal Medicine I, Gastroenterology, Hepatology, Endocrinology & Metabolism, Medical University of Innsbruck, Innsbruck, Austria; ^3^Department of Pediatric Gastroenterology, Nephrology and Metabolic Diseases, Charité Universitätsmedizin Berlin, Berlin, Germany; ^4^RNA Biology, Ethris GmbH, Planegg, Germany; ^5^Department of Medicine II, University Hospital, Ludwig-Maximilians-University Munich, Munich, Germany; ^6^Department of Pathology, Institute of Pathology, Ludwig-Maximilians-University, Munich, Germany; ^7^Department of Medicine and Immunology, University of Colorado Denver, Aurora, CO, United States

**Keywords:** TGF-β, Smad3, liver inflammation, serum levels, IL-37

## Abstract

**Background and Aims:** Chronic inflammation induces liver fibrosis, cirrhosis and potentially liver cancer. Kupffer cells modulate hepatic stellate cells by secreting immunologically active proteins as TGF-β. TGF-β promotes liver fibrosis *via* the activation of Sma- and Mad-related protein 3. IL-37 broadly suppresses innate and adaptive immune responses. Intracellular IL-37 interacts with Smad3. We hypothesize that IL-37 downregulates the activation of hepatic Kupffer and stellate cells and interferes with the TGF-β signaling cascade to modulate liver fibrogenesis.

**Methods:** The role of IL-37 on liver inflammation and fibrogenesis was assessed in three mouse models as well as isolated Kupffer- and stellate cells. Serum IL-37 was tested by ELISA in a clinical cohort and correlated with liver disease severity.

**Results:** Transgene expression of IL-37 in mice extends survival, reduces hepatic damage, expression of early markers of fibrosis and histologically assessed liver fibrosis after bile duct ligation. IL-37tg mice were protected against CCl_4_-induced liver inflammation. Colitis-associated liver inflammation and fibrosis was less severe in IL-10 knockout IL-37tg mice. Spontaneous and LPS/TGF-β-induced cytokine release and profibrogenic gene expression was lower in HSC and KC isolated from IL-37tg mice and IL-37 overexpressing, IL-1β stimulated human LX-2 stellate cells. However, administration of recombinant human IL-37 did not modulate fibrosis pathways after BDL in mice, LX2 cells or murine HSCs. In a large clinical cohort, we observed a positive correlation of serum IL-37 levels with disease severity in liver cirrhosis.

**Conclusions:** Predominantly intracellular IL-37 downregulates liver inflammation and fibrosis. The correlation of serum IL-37 with disease severity in cirrhosis suggests its potential as a novel target modulating the course of liver fibrosis.

## Introduction

Liver fibrosis and end stage cirrhosis represent the final pathway of chronic liver diseases and still lack a specific therapeutic approach (1). Whereas, chronic inflammation has been shown to promote hepatocarcinogenesis, the molecular link between inflammation and hepatic fibrogenesis has not been unraveled thoroughly (2). At the cellular level it was shown that Kupffer cells (KC) are the predominant inflammatory cells activating hepatic stellate cells (HSC). At the molecular level TGF-β is the core cytokine secreted by KCs to stimulate HSCs and to induce extracellular matrix (ECM) deposition (3). TGF-β promotes liver fibrosis and hepatocellular apoptosis through activation of Sma- and Mad-related protein 3 (Smad3) as a major TGF-β -signaling molecule (4, 5). Other cytokines such as IL-13, IL-17, and IL-33 were also shown to promote liver fibrogenesis by activating HSCs (6).

Cytokines of the IL-1 family (IL-1F) of ligands and receptors play a pivotal role in the modulation of immune responses (7). Recent data provide evidence for the role of IL-1F cytokine signaling in chronic liver injury and fibrosis (8, 9). For example, IL-1α and IL-1β are critically involved in the transformation of steatosis to steatohepatitis and liver fibrosis in hypercholesterolemic mice and ethanol-induced liver damage (10, 11). IL-33 promotes liver fibrosis through the induction of Th2 cells and attraction of innate lymphoid cells in fibrotic livers (12, 13).

IL-37 is a member of the IL-1 family and inhibits both innate and adaptive immunity by limiting the production of cytokines induced by IL-1 and Toll like receptors (TLR) (14, 15). IL-37 is a dual acting cytokine with extra- and intracellular targets of function. Extracellular IL-37 binds to IL-18 receptor alpha and single Ig IL-1R-related molecule (SIGIRR) (16, 17). Intracellular IL-37 translocates to the nucleus upon N-terminal processing by caspase-1 and binds to the TGF-β signaling molecule Smad3 (15–19).

Overexpression of IL-37 in cells of monocytic origin almost completely abolishes the production of proinflammatory cytokines in response to TLR-ligands or IL-1β. Vice versa, silencing of IL-37 in human PBMC increases the production of proinflammatory cytokines (15). IL-37tg mice are protected against LPS-induced endotoxemia (15), acute DSS-induced colitis (20) as well as obesity induced inflammation (21). We recently reported that transgene IL-37 suppresses colon carcinogenesis in chronic colitis (22). Wt mice treated with recombinant IL-37 (rhIL-37) are also protected in models of endotoxemia, acute lung injury, spinal cord injury, myocardial infarction, and asthma (2, 16, 23–26).

In the liver, IL-37 reduces inflammation induced by ischemia or concanavalin A-induced toxicity (27, 28). Although transgene IL-37 expression did not protect mice from liver injury in a model of binge drinking, rhIL-37 ameliorates hepatic inflammation and improves steatosis (29).

In humans, *IL-37* mRNA expression in the liver correlates with the body mass index of severely obese patients (30). Higher expression of IL-37 in hepatocellular carcinoma correlates with a better overall survival (31).

Here, we hypothesize that IL-37 not only suppresses liver inflammation but also modulates liver fibrosis by the interaction with Smad3. We tested the impact of IL-37 in three different models of liver fibrogenesis and dissected its function at the molecular and cellular level in hepatic Kupffer and stellate cells. Moreover, we demonstrate the correlation of serum IL-37 with disease severity in human cirrhosis.

## Materials and Methods

### Chemicals and Reagents

All reagents were purchased from Sigma-Aldrich GmbH (Munich, Germany) unless indicated.

### Animals

All animals received humane care and were acclimatized for 2 weeks before being studied. C57BL/6J mice expressing human IL-37 have been described previously (15). IL-10KO mice were obtained from Charles River Inc. (Boston, MA, USA). Animal protocols were approved by the review board of the Federal Government of Bavaria, Germany (Az. 55.2.1.54-2532-77-11), (Az. 55.2-1-54-2532-3-2017).

### Mouse Models of Liver Fibrosis

#### Bile Duct Ligation

Male wildtype, IL-37tg mice underwent ligation of the common bile duct under general anesthesia at the age of 6–8 weeks according to standard procedures (32). Control mice underwent sham operations in which the common bile duct was exposed but not ligated. Mice were either sacrificed 3 or 14 days after bile-duct ligation. In a separate experiment C57BL/6 mice were injected with rhIL-37 (1 or 5 μg) or vehicle 1 h before bile duct ligation and once again on day 2.

#### Chemically Induced Liver Fibrosis

CCl_4_ (0.6 ml/kg in oil) or oil was administered twice weekly *via* intra-peritoneal injection into 6–8 weeks old female C57BL/6 or IL-37tg mice for 6 weeks as described (33).

#### Colitis Associated Liver Disease

We recently described the protective role of IL-37 against colon inflammation and carcinogenesis during chronic colitis in IL-10 KO mice (22). Livers of homozygous IL-10KO and IL-10KO/IL-37tg mice from this study were analyzed by histology for fibrosis and by qPCR for gene expression after a 6 months course of chronic colitis.

### Liver Histology

At the end of each experiment, mice were bled by intracardial puncture and subsequently sacrificed. Livers were removed, segments of the right lobe fixed in 4% PFA and embedded in paraffin for histological evaluation. Hematoxylin/eosin (H&E), Van-Giesson, Sirius red, Mac2 (Cederlane Labs, Burlington, Canada) and CD3 (Zytomed Systems, Berlin, Germany) staining of 5 μm liver sections were performed according to standard protocols. Sirius red, Mac2-macrophage and CD3 T-lymphocyte quantification was performed by averaging the percentage of positive staining per area or number of positively stained cells in four randomly chosen HPF. Signs of inflammation and fibrosis were evaluated according to standardized HAI scoring system (34) and independently evaluated by a blinded pathologist (D. M.).

### Isolation of mHSC and KC

Quiescent hepatic stellate cells were isolated from Wt and IL-37tg mice (C57BL/6 background) according to standard methods as described in (35). During culture HSC show spontaneous differentiation into myofibroblasts and secrete IL-6 (see Supplementary Figure 1). Isolated cells were resuspended in 10 ml culture media (DMEM low glucose) and plated in 6-well TC plates with or without rhIL-37 (10, 100, or 1,000 ng/ml). Media and rhIL-37 were replaced every 2 days and tested for spontaneous IL-6 secretion by Elisa. In addition, cells were stimulated with LPS (100 ng/ml) on day 8 and TGF-β1 (100 pg/ml) on day 9. RNA analysis was performed on day 2, 6 h after LPS stimulation.

Murine Kupffer cells were isolated according to the same process as described above, though after gradient centrifugation cells appearing as a white milky colored ring were gently aspirated, added to 50 ml GBSS/B and centrifuged at 45 × g at 4°C for 2 min. Supernatant was carefully transferred to a fresh falcon and centrifuged at 4°C for 5 min at 700 × g. Cell pellet was resuspended in cell culture media plated for cell staining or migration assay (35).

### Tissue Culture

LX2 cells were obtained from Sigma Aldrich (SCC064) and routinely tested for mycoplasma contamination. For IL-37 overexpression experiments LX2 cells were plated in starvation media (DMEM + 0.5% FCS). Cells were transfected with 1 μg human IL-37 encoding chemically modified RNA or control using Lipofectamine RNAiMax. Subsequently, cells were stimulated with IL-1β (1 ng/ml). Chemically modified RNAs were kindly provided by Ethris GmbH (Planegg, Germany). For recombinant human IL-37 treatment LX2 cells were plated in starvation media (DMEM + 0.5% FCS) and exposed to a range of rhIL-37 for 24 h. Subsequently, cells were stimulated with IL-1β (1 ng/ml). Total RNA was collected 6 h after stimulation. Supernatant was tested for IL-6 by Elisa 24 h after stimulation.

### Cytokine Measurement

IL-6 was measured by ELISA (BD Biosciences, Heidelberg, Germany). CCL2, CCL4, IL-10, IL-13, Rantes, KC, G-CSF, IL12p40 were analyzed by BioplexAssay (Biorad, Munich, Germany). Serum IL-37 was determined with IL-37 (human) ELISA Kit (AdipoGen, Liestal, Switzerland) according to the manufacturer's specification.

### Serum Biochemistry

Serum samples were diluted 1:4 in PBS for determination of bilirubin, GPT, GOT, γGT, and alkaline phosphatase by routine methods.

### RNA Isolation and Quantification

Total RNA was isolated from 30 mg of snap frozen liver tissue or cell pellets using RNeasy Mini Kit (Qiagen, Hilden). RNA samples (1 μg) were reverse transcribed using SuperScript™ II Reverse Transcriptase (Invitrogen, Carlsbad, CA, USA). Gene expression levels were measured by quantitative PCR (SYBR Green Supermix, Biorad). Gene specific primers were designed using PrimerExpress and ordered from Eurofins MWG (Ebersberg, Germany) with purification grade HPLC. qRT-PCR reactions were performed in triplets in a 96-well format (BioRad iCycler). Fold changes of mRNA expression were calculated and if not stated otherwise normalized to Rpl13a gene expression using the ΔΔCt-method (36). Gene specific primers used are listed in Supplementary Table 1.

### Protein Isolation and Western Blotting

Cell pellets were resuspended in Pierce IP lysis buffer (Thermo Fisher Scientific, Munich, Germany) containing protease inhibitors. Ten milligram of liver tissue was homogenized in lysis buffer (PBS + 0.1% Triton). Protein quantification was performed using the BCA kit from Thermo Fisher. Between 5 and 35 μg of protein samples were loaded into each lane of an any kD Mini-PROTEAN TGX Precast Protein Gel and subsequently transferred to a PVDF membrane. After blocking (5% skim milk in PBS/Tween 20 0.05%) the membrane was probed with antibodies against Icam1 (R&D Systems, Abingdon, UK) and αSMA (Abcam, Cambridge, UK). β-actin (Cell signaling, Frankfurt, Germany) served as a loading control.

### Patients

We included 286 patients (84 female, 202 male) with liver cirrhosis and 22 healthy volunteers (8 female, 14 male) at the University Hospital of Innsbruck, Austria, in this study. Cirrhosis was confirmed by abdominal computer tomography and indirect cirrhosis signs, including esophageal varices, portal hypertension, ascites, hepatic encephalopathy, and thrombocytopenia. Model of end stage liver disease (MELD) score and Child Pugh (CP) score were calculated and the cohort size distributed within the ranges of CP scores were as followed (see Supplementary Table 2): 151 patients were diagnosed with CP score A, in 71 patients the calculated CP score was B and 64 patients fitted the criteria for CP C. Preexisting chronic liver disease was alcoholic liver disease in 143 patients and metabolic associated liver disease in 70 patients. Nine patients with chronic hepatitis B, 38 patients with chronic hepatitis C, and two patients with hepatitis D were included in the study. Furthermore, 11 patients with primary biliary cholangitis, two patients with secondary sclerosing cholangitis, one patient with primary sclerosing cholangitis and five patients with autoimmune hepatitis participated in this study. Five patients suffered from hereditary liver diseases: two patients diagnosed with Wilson's disease, two patients with alpha-1-antitrypsin deficiency and one patient with hemochromatosis. Ascites was detected by abdominal ultrasound. Two experienced physicians (each >3,000 US-exams) performed the US-examinations with the Philips EPIQ 5® (Philips Corporation, Amsterdam, The Netherlands). Hepatic encephalopathy was diagnosed by using the West Haven criteria in combination with the Psychometric Hepatic Encephalopathy score as described elsewhere (37). The study protocol was approved by the institutional ethics commission with an amendment to AN2017-0016 369/4.21.

#### Statistical Analysis

Data were expressed as mean ± standard error of mean or as median with first and third quartiles. For comparing quantitative variables, the Student's *t*-test or the non-parametric Mann–Whitney *U* or Wilcoxon signed-rank test were used as appropriate. Normality of distribution was determined by Kolmogorov-Smirnov test. The correlation analysis was estimated using the Spearman's p coefficient A *p* < 0.05 was considered as statistically significant. All statistical analyses were performed using SPSS Statistics v.22 (IBM, Chicago, IL) and GraphPad Prism 5 and 8 Version 8.4.2 for Macintosh (La Jolla, CA).

## Results

### Transgene IL-37 Expression Is Associated With Improved Survival, Liver Function Tests and Reduced Liver Fibrogenesis After BDL

**Figure 1:**
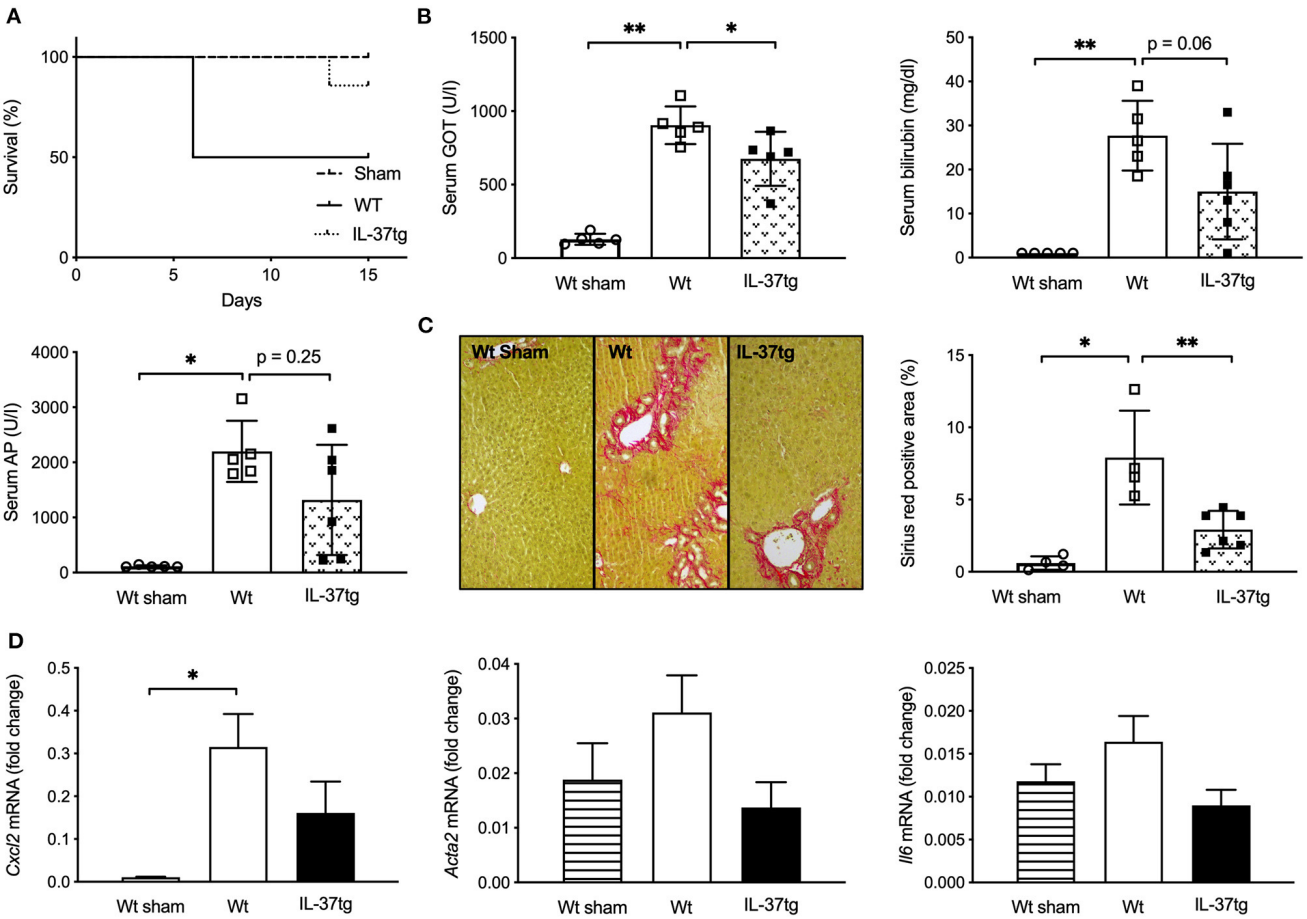
Transgene IL-37 expression is associated with improved survival, liver function tests, and reduced liver fibrogenesis after BDL. Wt and IL-37tg mice underwent BDL or sham operation. **(A)** Survival curve. **(B)** Liver function tests. **(C)** ECM deposition in liver sections as assessed and quantified by Sirius red staining. **(D)** Hepatic mRNA was quantified by qPCR 3 days after BDL. Fold changes of mRNA expression were calculated using the ΔΔCt-method normalized to *Rpl13a* gene expression. Open circles: Wt-sham (*n* = 5), Open boxes: Wt (*n* = 5), Closed boxes: IL-37tg (*n* = 6). *p < 0.05, **p < 0.01.

Since there is a broad range of pathologies inducing liver fibrosis we tested the role of IL-37 in different disease models. To investigate the impact of IL-37 in liver fibrosis induced by obstructive cholestasis, we performed bile duct ligation (BDL) in Wt and IL-37tg mice. By day 6, 5/10 Wt mice unexpectedly died (*n* = 2) or had to be sacrificed due to a high morbidity score (*n* = 3). Only one IL-37tg mouse had to be sacrificed on day 13 due to significant loss of body weight (Figure 1A). All sham-operated mice survived.

GOT, GPT, total serum bilirubin and alkaline phosphatase (AP) were normal in sham-operated mice and significantly higher in Wt mice after 15 days of BDL. IL-37tg mice had lower GOT levels compared to Wt mice after bile duct ligation and there was a trend of reduced serum bilirubin (45.8% reduction, *p* = 0.06) and AP (40.1% reduction, *p* = 0.25) (Figure 1B; Supplementary Figure 2A).

Fifteen days after BDL IL-37tg mice showed less liver fibrosis and less collagen deposition as determined by Sirius red staining in comparison to Wt mice (Figure 1C). Hepatic infiltration of Mac2-positive cells after BDL was similar in IL-37tg and Wt mouse livers despite an increase in comparison to sham-operated mice (Supplementary Figure 2B). Numbers of CD3-positive lymphocytes were slightly decreased in IL-37tg mice (27% reduction, *p* = 0.17, Supplementary Figure 2C).

In accordance to histologically overt liver fibrosis, *Cxcl2* gene expression as an early marker of liver fibrosis 3 days after BDL was significantly higher in Wt compared to sham-operated mice (Figure 1D). Transgene IL-37 expression was associated with lower, but not significantly reduced, gene expression levels of *Cxcl2, Acta2* (55.9% reduction) and *Il6* (45.1% reduction) compared to Wt mice and showed similar levels to sham operated mice (Figure 1D). Even though not significantly, *Col1a1, Tgf*β, and *Tnf*α were also lower in IL-37tg mice. There was no difference in *Timp1* expression (Supplementary Figure 3A).

### Recombinant IL37 Protein Does Not Downregulate Early Markers of Liver Fibrogenesis After BDL

**Figure 2:**
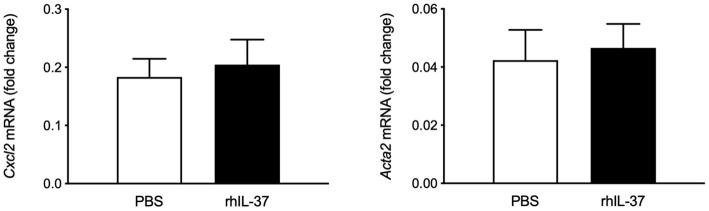
Recombinant IL37 protein does not downregulate early markers of liver fibrogenesis after BDL. Wt mice underwent bile duct ligation. Five microgram rhIL-37 (*n* = 12) or PBS (*n* = 14) was i.p. injected prior to BDL and the morning after. Hepatic levels of mRNA were quantified by qPCR after 3 days. Fold changes of mRNA expression were calculated using the ΔΔCt-method normalized to *Rpl13a* gene expression.

Intraperitoneal administration of rhIL-37 has been reported to downregulate ischemia-induced liver damage (28). To assess whether i.p. administered rh-IL-37 is sufficient to modulate early fibrosis markers after BDL, Wt mice were injected with increasing doses of rhIL-37 or vehicle prior to BDL and the day after. Gene expression analysis of *Cxcl2, Acta2* (Figure 2), *Col1a1* and *Timp1* (Supplementary Figure 3B), showed no difference between vehicle and rhIL-37-treated mice [1 μg (data not shown) or 5 μg of rhIL-37 per dose].

### Transgene IL-37 Expression Reduces CCl_4_-Induced Liver Inflammation

**Figure 3:**
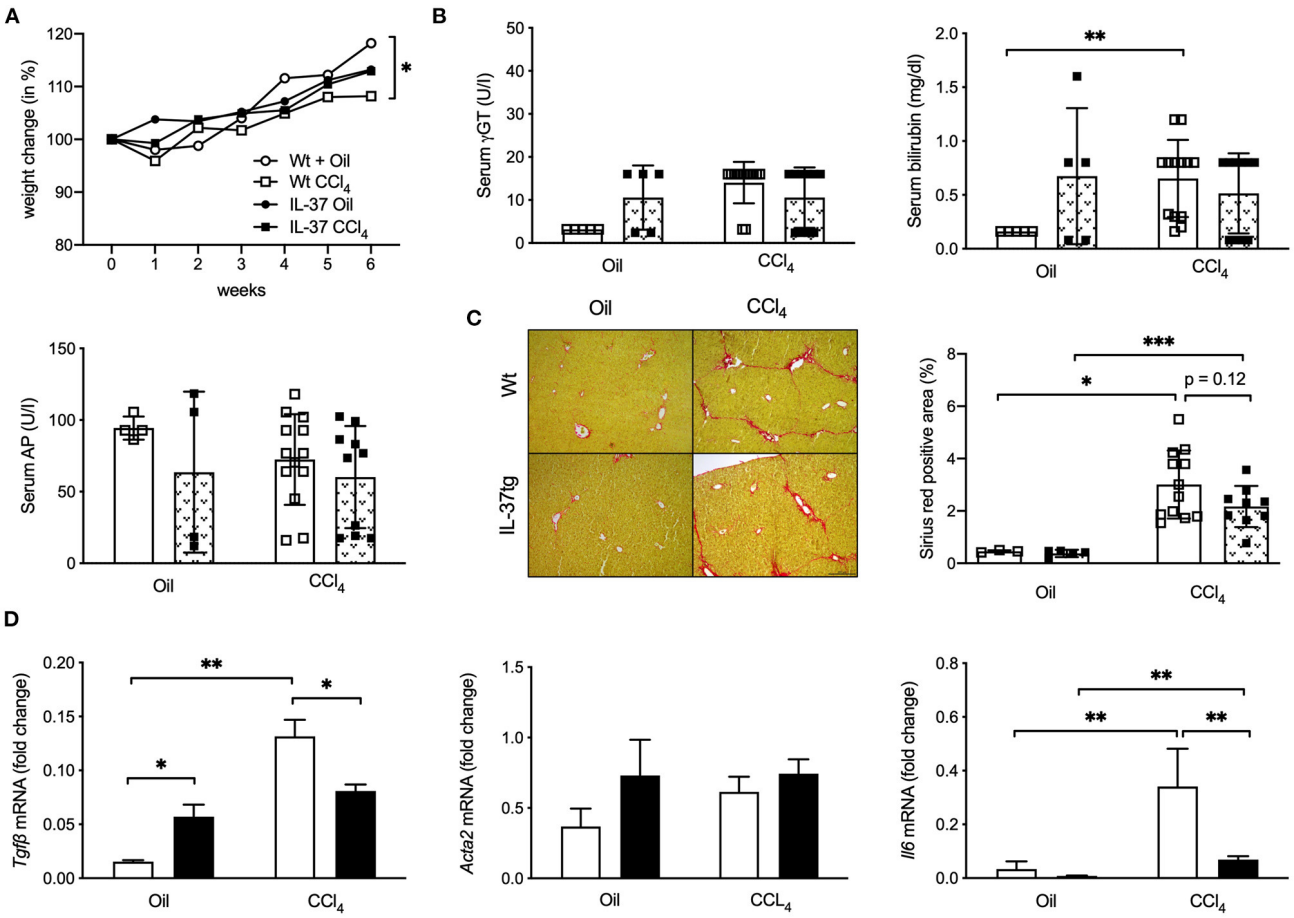
Transgene IL-37 expression reduces CCl_4_-induced liver inflammation. Wt and IL-37tg mice were injected with CCl_4_ or oil for 6 weeks. **(A)** Weight change **(B)** Liver function tests. **(C)** ECM deposition was assessed and quantified by Sirius red staining. **(D)** Hepatic mRNA levels were quantified by qPCR. Fold changes of mRNA expression were calculated using the ΔΔCt-method normalized to *Rpl13a* gene expression. Open boxes: Wt (Oil: *n* = 5, CCl_4_: *n* = 13), closed boxes: IL-37tg (Oil: *n* = 5, CCl_4_: *n* = 10). *p < 0.05, **p < 0.01, ***p < 0.001.

In addition to BDL as a model of obstructive cholangiopathy and liver fibrosis, we evaluated the effect of transgene IL-37 in CCl_4_-induced toxic liver injury and consecutive liver fibrosis. After a 6 week course of CCl_4_-injections Wt mice showed a reduced body weight compared to controls, while IL-37tg mice showed no significant weight loss (Figure 3A). No significant differences were observed for γGT (Figure 3B). Overall bilirubin levels were low, however serum bilirubin was higher in CCl_4_ treated Wt mice compared to control and there was no difference in treated and untreated IL-37tg mice (Figure 3B). AP was comparably low in both oil and CCl_4_-treated IL-37tg mice (Figure 3B). Quantification of collagen deposition as assessed by Sirius-red-staining showed an increase in Wt and IL-37tg mice compared to oil treated mice (Figure 3C). A slightly lower collagen deposition was observed in livers of IL-37tg mice (27.9% reduction, *p* = 0.12). IL-37tg livers showed higher baseline *Tgf*β mRNA levels but significantly lower levels after CCl_4_ treatment (Figure 3D). There was no difference in *Acta2* mRNA levels between the groups (Figure 3D). Hepatic *Il6* mRNA concentration was increased by CCl_4_ treatment but significantly lower in IL-37tg compared to Wt mice (Figure 3D).

### Transgene IL-37 Expression Reduces Colitis Associated Liver Inflammation and Fibrosis

**Figure 4:**
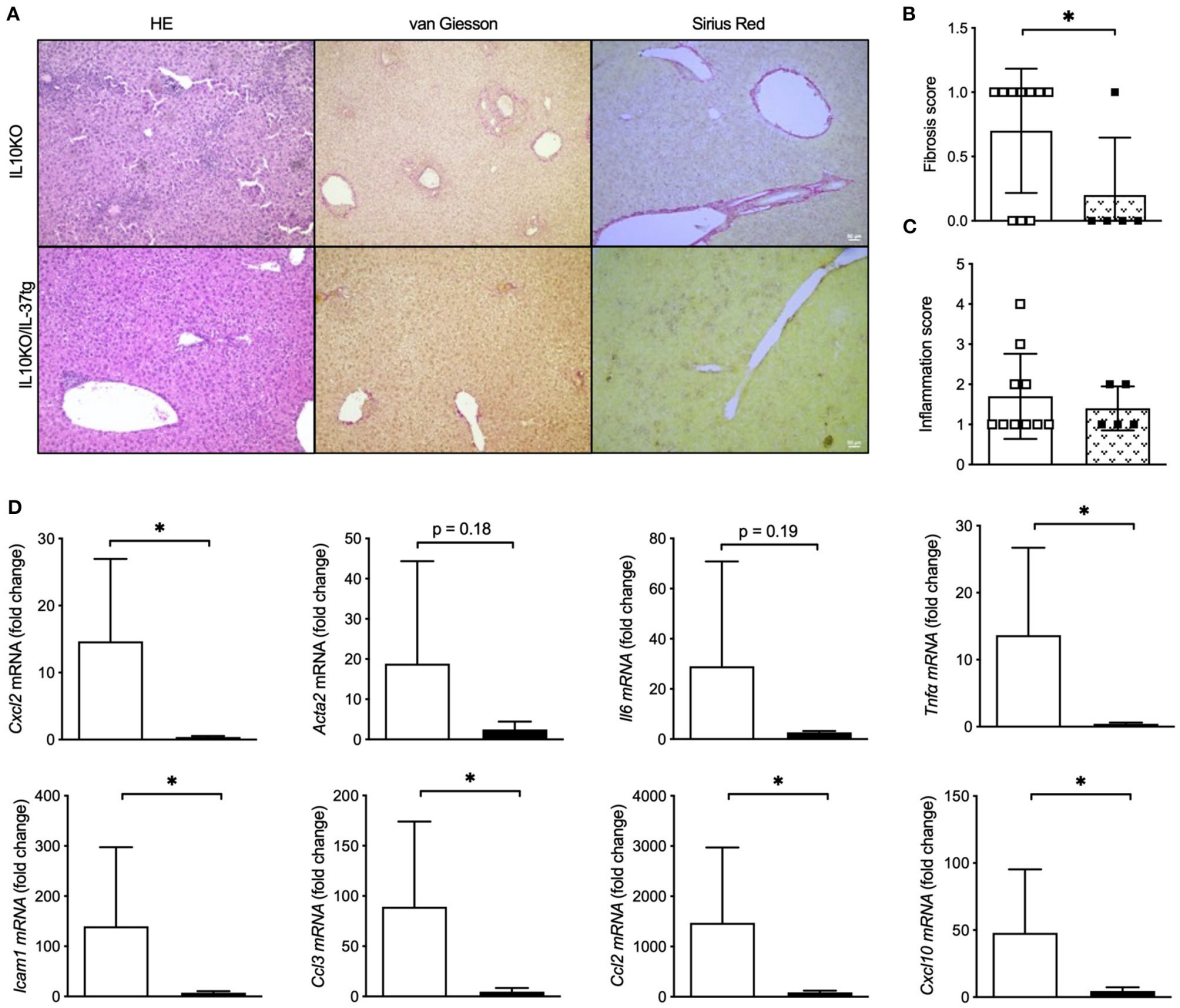
Transgene IL37 expression reduces colitis associated liver inflammation and fibrosis. Livers of homozygous IL-10KO and IL-10KO/IL-37tg mice were analyzed after a 6 month course of chronic colitis. **(A)** Liver histology, **(B)** liver fibrosis and **(C)** liver inflammation was assessed using the HAI scoring system. **(D)** Hepatic mRNA levels were quantified by qPCR. Fold changes of mRNA expression were calculated using the ΔΔCt-method normalized to *TBP* gene expression. Open boxes/column: IL-10KO (*n* = 5), closed boxes/column: IL-10KO/IL-37tg (*n* = 10), *p < 0.05.

As a third model we evaluated whether IL-37tg expression reduces liver inflammation and fibrosis in the IL-10KO mouse model of chronic colitis which we recently published (22). IL-10KO mice showed mild liver inflammation and fibrosis with the age of 6 months in the course of chronic colitis. IL-10KO/IL-37tg mice were protected from colon carcinogenesis (22) and, here, show reduced liver fibrosis (Figures 4A,B), though histologic liver inflammation (Figure 4C) did not differ to IL-10KO mice. Hepatic gene expression of proinflammatory *Cxcl2, Acta2, Il6, Tnf*α*, Icam1, Ccl3, Ccl2*, and *Cxcl10* was downregulated in IL-10KO/IL-37tg mice (Figure 4D).

### IL-37 Overexpression Reduces the Pro-inflammatory Response of Human LX2 Stellate Cells

**Figure 5:**
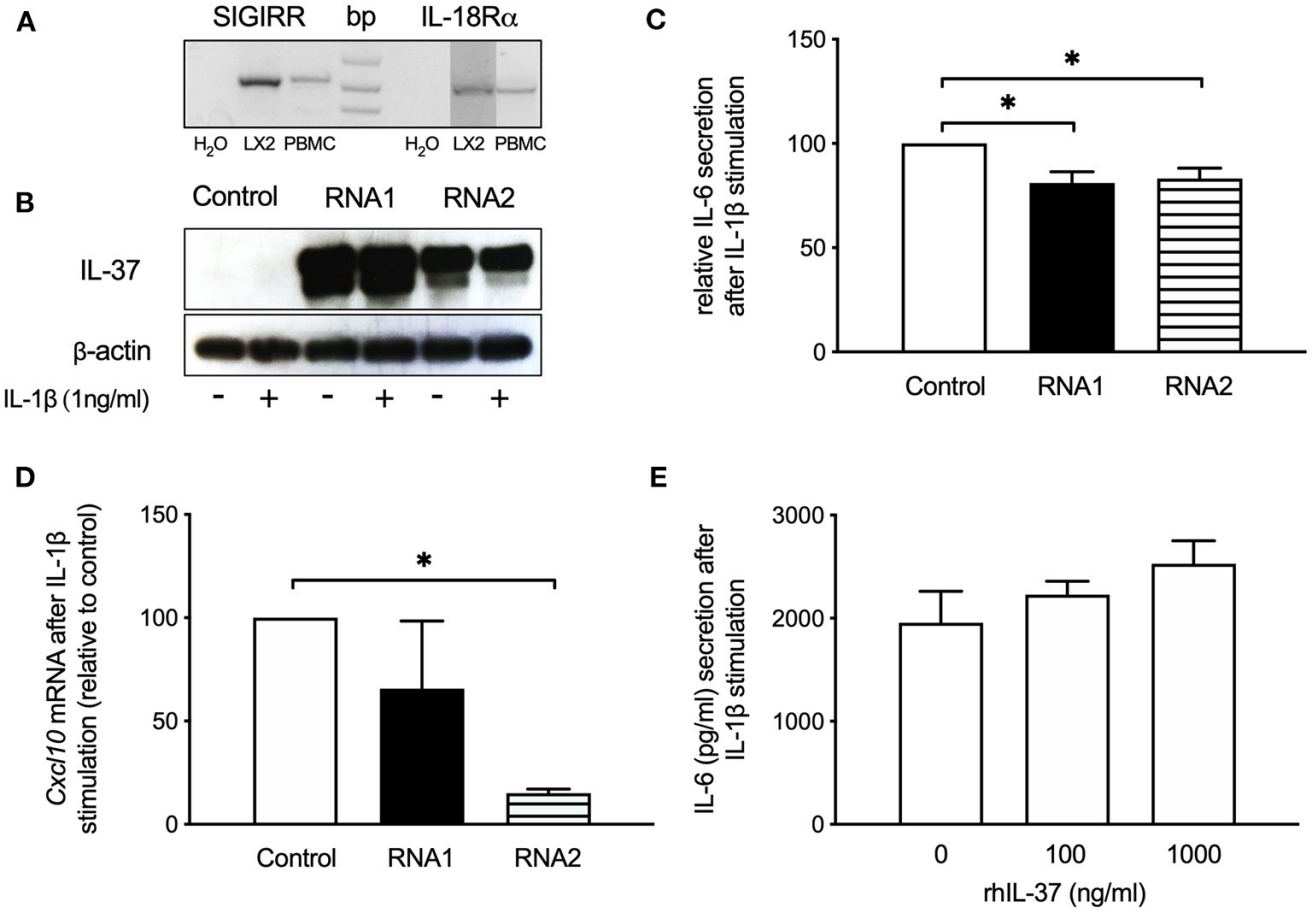
IL-37 overexpression reduces the pro-inflammatory response of LX2 stellate cells. LX2 cells were transfected with two different IL-37 expressing cmRNA or vehicle or exposed to rhIL-37 protein. Cells were stimulated with IL-1β (1 ng/ml) 24 h after transfection or treatment with rhIL-37. **(A)** IL-37 receptor expression. *SIGIRR* and *IL-18R*α mRNA from LX2 and human PBMC was detected by RT-PCR. **(B)** Lysates of LX2 cells transfected with IL-37 cmRNA1, cmRNA2 or control mRNA were analyzed for IL-37 protein expression by western blot. **(C)** IL-6 in supernatants of IL-37 mRNA-transfected LX2 cells after IL-1β-stimulation. **(D)** mRNA levels by qPCR 6 h after IL-1β-stimulation and are expressed relative to control mRNA transfection. **(E)** IL-1β-stimulated LX2 cells treated with rhIL-37. IL-6 in cell supernatants was measured after 24 h stimulation. *n* = 3, *p < 0.05.

We next tested whether overexpression of IL-37 reduces the inflammatory response of human LX-2 stellate cells. After confirming that LX2 stellate cells express the IL-37 receptor SIGIRR and IL-18Rα (Figure 5A), we overexpressed IL-37 in LX2 cells by transfection with liposomal-coated, chemically modified RNAs (Figure 5B). We tested two different preparations of IL-37-expressing cmRNAs. Cells overexpressing IL-37 showed reduced IL-6 secretion (RNA1: 19% reduction, RNA2: 17% reduction) upon stimulation with IL-1β (Figure 5C). *Cxcl10* mRNA expression was reduced by 85% by cmRNA2 (Figure 5D). In contrast, increasing concentrations of rhIL-37 protein did not downregulate IL-6 secretion from LX-2 cells upon stimulation with IL-1β (Figure 5E).

### Transgene IL-37 Expression Reduces the Pro-inflammatory Response of mHSC and KC

**Figure 6:**
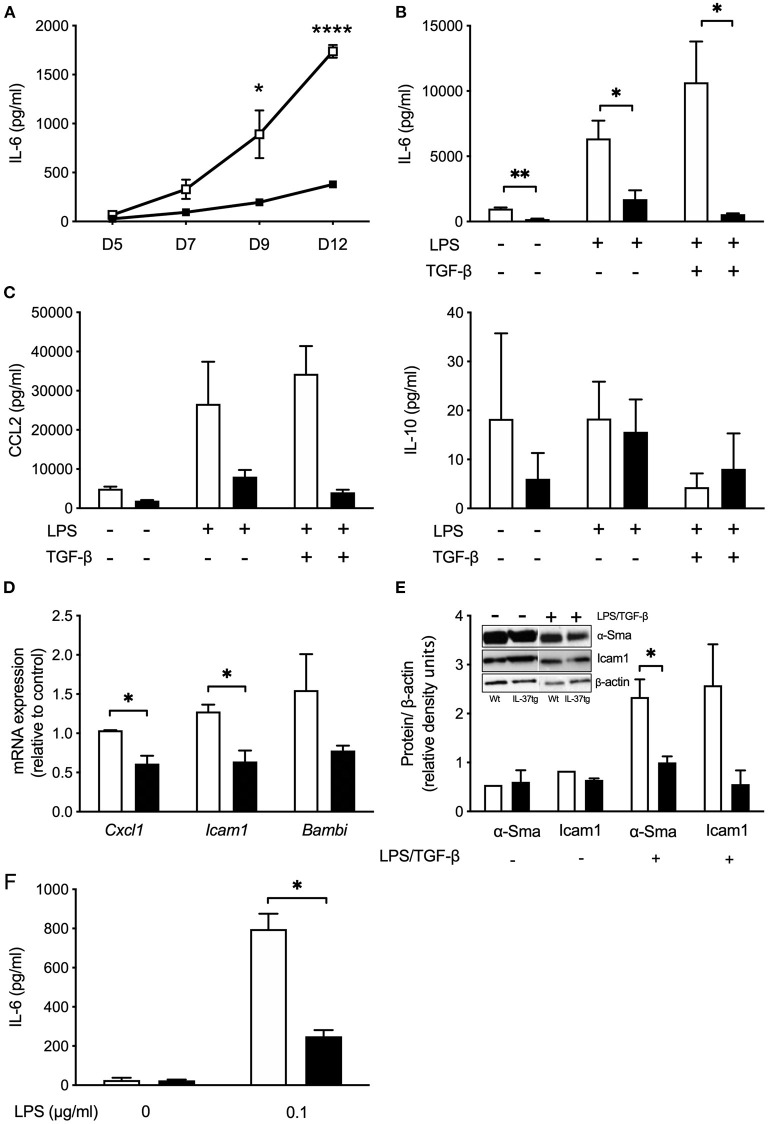
Transgene IL-37 expression reduces the pro-inflammatory response of mHSC and KC. **(A)** Supernatants of freshly isolated murine HSC were collected on day 5, 7, 9, and 12 during myofibroblast differentiation and tested for spontaneous IL-6 release. **(B)** Cultured HSC were stimulated with LPS (100 ng/ml) on day 8, and TGFβ (100 pg/ml) on day 9. Cells were harvested on day 12 and supernatant tested for IL-6 or CCl2 and IL-10 **(C)**. **(D)** Freshly isolated mHSC were cultured overnight and stimulated with LPS (100 ng/ml) the next day. Total RNA was collected 6 h after stimulation and analyzed by qPCR. Fold changes of mRNA expression (relative to control) were calculated using the ΔΔCt-method normalized to *Rpl13a* gene expression. **(E)** HSC were stimulated as described in **(B)** and Icam1 and α-Sma was analyzed in cell lysates (day 12) by western blotting. **(F)** Freshly isolated KC were stimulated with LPS (0.1 μg/ml). Supernatants were tested for IL-6 24 h after stimulation. HSC or KC were isolated from Wt (open boxes/bars) or IL-37tg mice (closed boxes/bars). *n* = 3, *p < 0.05, **p < 0.01, ****p < 0.0001.

Hepatic stellate cells are the main collagen producing cell type in liver fibrosis. To further evaluate the role of IL-37 in primary stellate cells we isolated HSCs from Wt and IL-37tg mouse livers. Spontaneous IL-6 secretion from HSC over a 12 days period in culture was markedly lower in HSC isolated from IL-37tg mice compared to Wt HSCs (Figure 6A). *In vitro* differentiated IL-37tg HSC released less IL-6 in response to LPS and LPS plus TGF-β (Figure 6B). CCL2 was lower by trend in supernatants of IL-37tg HSC (*p* = 0.1) but there was no difference in IL-10 (Figure 6C). After LPS stimulation *Cxcl1* and *Icam1* mRNA was significantly lower in IL-37tg HSC. *Bambi* showed a trend of reduction in IL-37tg HSC (Figure 6D). α-Sma protein expression was lower in IL-37tg mHSC after LPS/TGF-β (Figure 6E). Icam1 protein was also reduced but without statistical significance (*p* = 0.1). Unstimulated Wt or IL-37tg HSC showed no difference in α-SMA and Icam1 protein expression (Figure 6E).

Similar to IL-37tg HSC, KC isolated from IL-37tg mice secrete less IL-6 after LPS (Figure 6F).

### Recombinant IL-37 Does Not Modulate the Pro-inflammatory Response of HSC

**Figure 7:**
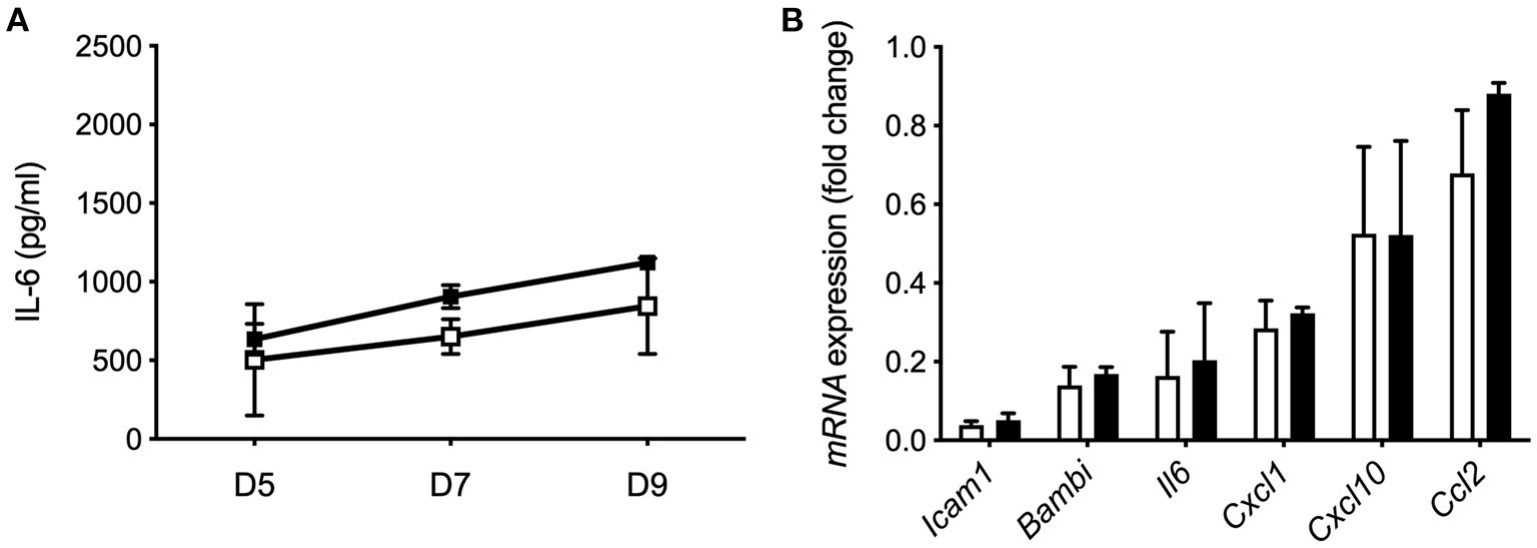
Recombinant IL-37 protein does not modulate the pro-inflammatory response of HSC. **(A)** Recombinant IL-37 (1 μg/ml) was added every second day along with fresh media to cultured Wt HSC. Supernatants were collected on day 5, 7, and 9 during myofibroblast differentiation and tested for spontaneous IL-6 release. **(B)** Freshly isolated mHSC from Wt mice were cultured with 1 μg/ml rhIL-37 overnight and subsequently stimulated with LPS (100 ng/ml). mRNA levels were measured by qPCR 6 h after stimulation. Fold changes of mRNA expression were calculated using the ΔΔCt-method normalized to *Rpl13a* gene expression. Open boxes/bars: PBS, closed boxes/bars: rhIL-37. *N* = 3.

To investigate whether extracellular IL-37 is sufficient to modulate spontaneous IL-6 secretion, we treated mHSC isolated from wt mice with rhIL-37 protein during culture. Increasing concentrations of rhIL-37 (10, 100, or 1,000 ng/ml) had no effect on spontaneous IL-6 secretion (Figure 7A) or LPS-induced pro-inflammatory gene expression in HSC (Figure 7B).

### Migration of KC Toward HSC Is Not Modulated by IL-37

Activated KC migrate toward HSC to stimulate collagen deposition. We therefore tested whether IL-37tg expression modulates the migration of KC toward the supernatant of LPS-stimulated Wt HSC. Neither transgene IL-37 expression in KC nor the supernatant of IL37tg HSC modulated migration of KC *in vitro* (Table 1).

**Table 1:** Migration of KC toward HSC is not modulated by IL-37.

**Migration index**	**Wt KC**	**IL-37tg KC**
SN Wt mHSC	97.81 ± 21.09	97.47 ± 6.70
SN IL-37tg mHSC	89.24 ± 5.42	101.5 ± 3.82

*Kupffer cells from Wt and IL-37tg were isolated from mouse livers (n = 3, one liver per experiment) and placed in the upper chamber of a Boyden Chamber Migration assay. Supernatants from LPS and TGF-β stimulated Wt or IL-37tg mHSCs were placed in the lower chamber. Migration index was assessed after 8 h*.

### MELD Score and Child-Pugh Score Correlate With Serum IL-37

**Figure 8:**
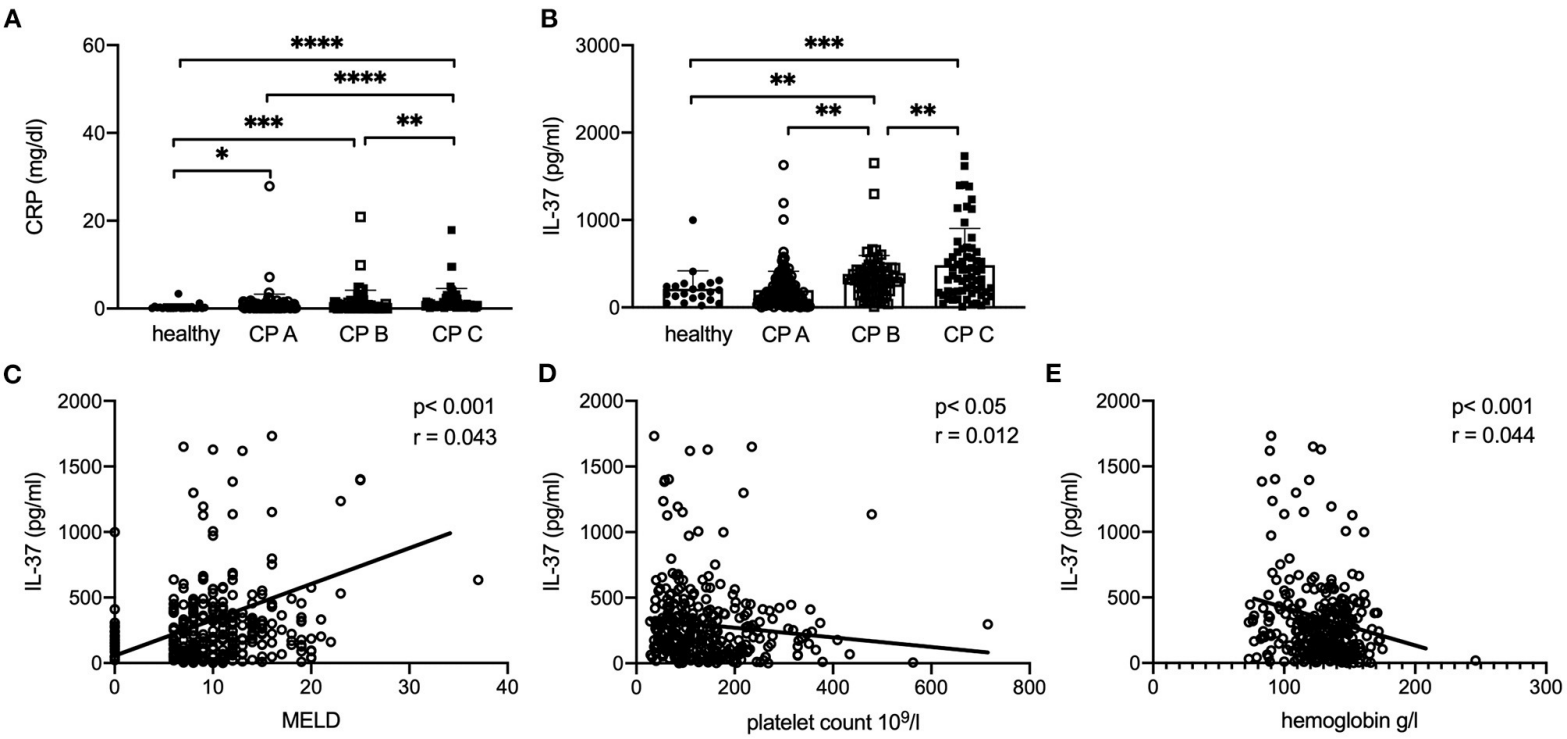
Serum levels of IL37 correlates with disease severity in patients with liver cirrhosis. Correlation of CRP **(A)** and serum IL-37 **(B)** with CP score in patients with liver cirrhosis compared to healthy control patients. **(C)** MELD score in patients with cirrhosis compared to healthy controls (MELD in healthy controls was assumed 0). Correlation of serum IL-37 with platelet count **(D)** and hemoglobin **(E)** in patients with cirrhosis and healthy controls. ****p < 0.0001, ***p < 0.001, **p < 0.01, *p < 0.05: according to Spearman correlation or Student's *t*-test.

Both CRP and IL-37 serum levels correlate with Child Pugh (CP) score and are significantly lower in healthy controls than in patients with different extent of liver fibrosis as indicated by Child-Pugh-score (Figures 8A,B). Furthermore, IL-37 levels showed significant differences between CP A, CP B, and CP C patients (*p* < 0.01, Figure 8B). A positive correlation was observed between MELD score and IL-37 levels (*p* < 0.001, r = 0.043, Figure 8C). Likewise, platelet count and hemoglobin values correlated negatively with IL-37 in cirrhosis patients (*p* < 0.05, r = 0.012, Figure 8D; *p* < 0.001, r = 0.044, Figure 8E). IL-37 did not correlate with levels of C-reactive protein, albumin, INR, liver transaminases, gamma-glutamyltransferase, alkaline phosphatase, leukocytes and creatinine (data not shown).

## Discussion

Chronic inflammation is an important trigger of liver fibrogenesis. Although well-described, inflammatory pathways have received little attention as therapeutic targets for chronic liver diseases (2). IL-37 exerts broad-spectrum anti-inflammatory effects *in vitro* and *in vivo* (14) and interferes with the TGF-β signaling pathway by functional interaction with Smad3 (15–19). We therefore hypothesized that IL-37 downregulates the activation of hepatic Kupffer and stellate cells and also modulates liver fibrogenesis by functional interaction with the TGF-β signaling pathway. We show that IL-37 improves the clinical outcome and downregulates liver inflammation and fibrogenesis in mice as well as the activation of Kupffer- and stellate cells. In addition, we demonstrate the correlation of IL-37 serum levels with disease severity in human liver cirrhosis.

Obstructive cholestasis in patients induces the release of serum transaminases, alkaline phosphatase and bilirubin (38). Consistently, Wt and IL-37tg mice show elevated GOT, GPT, AP and bilirubin serum levels after BDL in this study. IL-37tg mice had lower GOT levels than Wt mice indicating less hepatocellular damage. In the model of chemically-induced liver fibrosis liver function tests were normal both in vehicle- or CCl_4_-treated Wt and IL-37tg mice. This reflected induction of mild liver disease by CCl_4_ as intended for the study (data not shown).

Mortality after BDL is described as being as low as 5% (32). Despite the fact that there was no perioperative mortality, 50% of Wt mice died or had to be sacrificed during the second week after BDL mainly due to weight loss and worsening clinical condition. However, IL-37tg expression was associated with markedly improved survival after BDL and only one IL-37tg mouse had to be removed from the experiment prematurely due to weight loss. Since all sham-operated mice survived without sequelae other factors than surgery, such as local microbiota, might have contributed to high morbidity and mortality rate in Wt mice after BDL. Weight loss is also well-described in CCl_4_-induced liver inflammation and fibrosis (37) and was similarly less in IL-37tg mice indicating an improved clinical condition by IL-37tg expression in the chemically-induced liver fibrosis model.

In parallel to the mitigated clinical outcome, IL-37tg mouse livers after BDL showed less fibrosis. In CCl_4_-induced liver injury we also observed a trend toward less liver fibrosis in IL-37tg mice.

As a third model we examined livers of IL-10KO and IL-10KO/IL-37tg mice during chronic colitis, since hepatobiliary involvement in IBD is common and affects 20–30% of patients with IBD (2, 39). Despite low-grade histologic liver inflammation, IL-37tg expression was associated with reduced histologically proven fibrosis.

Analyzing the immune cell infiltrate in livers after BDL we found that numbers of Mac2-positive hepatic macrophages were similar in IL-37tg and Wt mice. However, we observed a trend of lower numbers of CD3 positive lymphocytes in livers of IL-37tg mice indicating less hepatocellular inflammation.

When activated, liver infiltrating macrophages and T-lymphocytes secrete cytokines such as IL-6 and TGF-β to stimulate, in concert with KCs, the proliferation and activation of HSCs (40, 41). Since overexpression of IL-37 downregulates the proinflammatory response of immune cells *in vitro* and *in vivo* we hypothesized that proinflammatory mediators are also lower in livers of IL-37tg mice (14). Indeed, there was a trend of reduced expression of proinflammatory and profibrogenic genes in IL-37tg mouse livers in the early course after BDL. Similarly, in CCl_4_-induced liver fibrosis levels of hepatic *Il6* and *Tgf*β gene expression were markedly lower in IL-37tg mice. Most strikingly was the reduction of *Tnf*α*, Cxcl10*, and other proinflammatory and profibrogenic genes in livers of IL-37tg mice during chronic colitis suggesting that IL-37 modulates fibrosis both by inhibiting inflammation and downregulating fibrosis-inducing pathways.

Intraperitoneally injected rhIL-37 reduces ischemia/reperfusion-induced liver damage (28). However, in our model of BDL, systemic administration of rhIL-37 at different doses, acting by binding to the membrane receptor, was not sufficient to limit proinflammatory or profibrogenic gene expression at day 3 after BDL. Therefore, we speculate that intracellular IL-37, as expressed in IL-37tg mice, plays a more dominant role in modulating cholestasis-induced liver inflammation and fibrosis after BDL than extracellular IL-37. The effect of rhIL-37 protein could not be tested in CCl_4_-induced liver fibrosis or during chronic colitis due to the long-term nature of both models during which subcutaneous or i.p. injections would have induced an antibody response against human IL-37 protein.

The crosstalk between KC and HSC is crucial for the activation of HSC and the initiation of liver fibrogenesis. KC secrete proinflammatory cytokines in response to danger signals such as endotoxin (42). In turn, these cytokines activate and thereby initiate proliferation and myofibroblast differentiation of HSCs, which then produce components of ECM as well as adhesion molecules like α-SMA and Icam1 (43–45). Since IL-37 downregulates inflammation and was shown to have functional interaction with profibrogenic TGFβ-signaling molecule Smad3, we tested the impact of IL-37 on the function of human LX2 stellate cells and primary mouse KC and HSC.

When we overexpressed IL-37 in LX2 stellate cells by cmRNA we observed a reduction of IL-1β-induced IL-6 secretion and profibrogenic *Cxcl10* gene expression. However, despite expression of the IL-37 receptor, treatment of LX2 cells with rhIL-37 had no effect. We particularly paid attention to titrate concentrations of rhIL-37 down to as low as 10 ng/ml (data not shown), since it was shown that low concentrations of rhIL-37 are more effective to downregulate the inflammatory response of primary human macrophage cells *in vitro* than higher concentrations (16). The lack of response to rhIL-37 indicates that intracellular expression of IL-37, as induced by cmRNA transfection, is more effective to reduce the inflammatory response of LX-2 stellate cells than exogenously applied, extracellular rhIL-37. Similar results were obtained from mouse HSC-derived myofibroblasts, where only transgene IL-37 but not rhIL-37 protein reduced spontaneous, LPS or LPS/TGF-β-induced IL-6 secretion and pro-inflammatory gene expression.

Common markers of HSC activation such as Icam1 and α-Sma were also reduced in HSC-derived myofibroblasts isolated from IL-37tg mice after LPS or LPS/TGF-β stimulation. These results indicate that IL-37 overexpression reduces both the inflammatory response as well as fibrogenesis by HSC. Notably, there was no difference in IL-10 secretion from IL37tg and Wt HSCs. This stands in accordance with our previously published observation that the immunomodulatory function of IL-37 in macrophage cells or PBMC is not mediated by IL-10 (15, 18).

Activated liver HSC/myofibroblasts release a range of chemokines including CCL2, CCL3, and CXCL10 to attract lymphocytes (46). Previous studies have shown that IL-37 inhibits the formation of macrophage pseudopodia suppressing cell migration (15, 47). Our results show that HSC-derived myofibroblasts from IL-37tg mouse livers secrete slightly less KC attracting chemokine CCL2. We hypothesized that IL-37tg expression in HSC impairs migration of KC toward HSC. However, neither transgene expression of IL-37 in KCs nor the supernatant of stimulated IL-37tg HSC modulated the migration behavior of KC. Therefore, we speculate that modulation of KC migration toward HSC is unlikely to contribute to reduced liver fibrogenesis in IL-37tg mice.

At the molecular level we reported that intracellular IL-37 interacts with Smad3 to reduce inflammation (15). Smad3 itself is activated by phosphorylation at the C-terminus (pSmad3C) or at the linker domain (pSmad3L) through TGF-β type I receptor or TGF-β-dependent c-Jun N-terminal kinase (48). The pSmadC pathway inhibits growth of normal cells as a tumor suppressor, whereas pSmadL-mediated signaling promotes ECM deposition and subsequent fibrosis as well as tumor cell invasion (2, 49, 50). In a human HCC cell line, transfected IL-37 directly targets pSmad3L/c-myk signaling to suppress oncogenic pSmadL signaling and to promote tumor-suppressive pSmad3C signaling (48). In line with this observation, our yet unpublished, confocal microscopy data show that IL-37 colocalizes with pSmad3L in human fibrotic livers (MS in preparation). Moreover, Kim et al. recently reported that intranasally-administered IL-37 attenuates bleomycin-induced lung fibrosis in mice and is associated with lower TGF-β protein in lungs (51). Accordingly, we measured lower TGF-β mRNA levels in livers of IL-37tg mice after CCl_4_-treatment. Li et al. also showed that intranasal administration of a lentivirus expressing IL-37 improved survival, attenuated pulmonary inflammation and collagen deposition in bleomycin-treated mice (52). In summary, these reports lead us to speculate that, beside limiting the inflammatory response, IL-37 directly impacts HSC-mediated liver fibrogenesis by interacting with TGF-β dependent pathways.

As well-established in liver fibrosis we show that CRP, a surrogate parameter for inflammation, correlates with the CP score in our large cohort of patients with liver fibrosis. Interestingly, IL-37 serum levels are also higher in patients with liver cirrhosis correlating with the CP- and MELD score as well as platelet counts and hemoglobin levels. A similar phenomenon has been described for IL-1 receptor antagonist, another anti-inflammatory IL-1 family member (53). This might reflect the response of the host to fight against overwhelming hepatic inflammation and consecutive fibrosis.

In summary, we show evidence that transgene expression of IL-37 reduces liver inflammation and fibrosis in BDL-, CCl_4_-, and colitis-associated liver disease in mice. We suggest that predominantly intracellular IL-37 modulates liver fibrosis in two definite ways. Firstly, the interaction of IL-37 with pSmad3L directly targets the fibrotic pathway. Secondly, IL-37 downregulates liver inflammation and subsequent HSC activation by limiting the release of proinflammatory and profibrogenic cytokines from infiltrating lymphocytes, macrophages, and KC. Thus, IL-37-dependent mechanisms may represent a future target for the treatment of inflammatory and fibrosing liver diseases. The correlation of serum IL-37 with disease severity of liver cirrhosis in humans indicates the clinical relevance of our experimental findings. Further studies are needed to unravel the molecular mechanisms of IL-37 in liver fibrogenesis in more detail.

## Data Availability Statement

The raw data supporting the conclusion of this article is available in the Supplementary Material.

## Ethics Statement

The animal study was reviewed and approved by Federal Government of Bavaria, Germany.

## Author Contributions

SM, HN-P, ME, AR, SH, FR, HT, and PB contributed to planning and conduction of experiments. SM, LG, SH, DM, GD, FR, HT, CD, and PB contributed to evaluation and discussion of the results. SM, PB, and HT prepared the manuscript. All authors contributed to the article and approved the submitted version.

## Conflict of Interest

SH was employed by the company Ethris GmbH. The remaining authors declare that the research was conducted in the absence of any commercial or financial relationships that could be construed as a potential conflict of interest.

## Abbreviations

AP: alkaline phosphatase
BDL: Bile duct ligation
CCl_4_: Carbontetrachloride
CP: Child Pugh
ECM: Extracellular matrix
HAI: Hepatic Activity Index
HCC: hepatocellular carcinoma
HPF: High power field
IL-1F: Interleukin-1 family
KC: Kupffer cells
LPS: Lipopolysaccharide
(m)HSC: (murine) hepatic stellate cells
MELD: model of end stage liver disease
MMP: Matrixmetalloproteinase
NK: natural killer cells
rh: recombinant human
SIGIRR: single Ig IL-1R-related molecule
SN: Supernatant
TGF: Transforming growth factor
TIMP: Tissue inhibitor of metalloproteinase
TLR: Toll-like receptor
Wt: Wild type.
