# Supplementary material for: Modulation of Liver Inflammation and Fibrosis by Interleukin-37
Source: Front Immunol. 2021 Mar 4;12:603649. doi: 10.3389/fimmu.2021.603649 (PMC7970756; doi:10.3389/fimmu.2021.603649)
Supplement: Supplementary file 2 [file Data_Sheet_1.docx]

**Supplemental Figure 1: Spontaneous activation and myofibroblast activation of Wt murine HSC in culture.** Wt murine hepatic stellate cells were isolated according to standard procedures and differentiated to activated mHSC by culturing them for 15 days. (A) Cell culture supernatant was collected every second day and tested for spontaneous IL-6 release. (B) Cells were fixed on day 12 after isolation and stained for α-SMA as a marker for myofibroblast activation (C) α-Sma protein expression was determined by western blot analysis on 12 day after isolation. β-actin was run as loading control. N=1.

**Supplemental Figure 2: Transgene IL-37 improves survival and reduces fibrosis after BDL.** Wt and IL-37tg mice underwent BDL or sham operation. (A) Serum GPT, (B) hepatic infiltrate of Mac2 positive macrophages and (C) CD3 positive cells.

**Supplemental Figure 3: Early markers of hepatic inflammation and fibrogenesis in mice after BDL.** (A) Wt (n=10) and IL-37tg (n=8) mice underwent BDL or sham operation. (B) Wt mice underwent bile duct ligation. 5 µg rhIL-37 (n=12) or PBS (n=14) was i.p. injected prior BDL and the morning after. Hepatic levels of mRNA were measured by qPCR after 3 days. Fold changes of mRNA expression were calculated using the ΔΔCt-method normalized to *Rpl13a* gene expression.

**Supplemental Table 1: Gene specific qRT-PCR primer**

| Gene | Forward Primer | Reverse Primer |
| --- | --- | --- |
| *Cxcl1* | *TGA AGG TGT TGC CCT CAG G* | *AAC CAA GGG AGC TTC AGG GT* |
| *Icam1* | *CGC ACA GAA CTG GAT CTC AGG* | *TTT GGG ATG GTA GCT GGA AGA* |
| *Bambi* | *CCA GCT ACT TCT TCA TCT GGC* | *GAT CTC TCC TTT GGT GAG CAG* |
| *Il6* | *ATT ACA CAT GTT CTC TGG G* | *GGA CTC TGG CTT TGT CTT* |
| *Tgfβ* | *GCC AAC TTC TGT CTG GGA CC* | *CCG GGT TGT GTT GGT TGT AGA* |
| *Cxcl2* | *ACC AAC CAC CAG GCT ACA GG* | *CTC AAG CTC TGG ATG TTC TTG AAG* |
| *Col1a1* | *CGG TAA CGA TGG TGC TGT T* | *CTT CAC CCT TAG CAC CAA CT* |
| *Acta2* | *CCA TCT TTC ATT GGG ATG GAG* | *TAG CAT AGA GAT CCT TCC TGA* |
| *Tnfα* | *AGG CGG TGC CTA TGT CTC AG* | *GAC CGA TCA CCC CGA AGT T* |
| *Cxcl10* | *CTG GGT CTG AGT GGG ACT CAA* | *TTC CCT ATG GCC CTC ATT* |
| *Ccl2* | *GTT GGC TCG CCA GAT GCA* | *TGA TCC TCT TTA GCT CTC CAG C* |
| *Ccl3* | *CCA TGA CAC TCT GCA ACC AAG T* | *GAT GAA TTG GCG TGG AAT CTT C* |
| *Tbp* | *GCC CGA AAC GCC GAA TAT* | *CCG TGG TTC GTG GCT CTC T* |
| *Rpl13a* | *ATC CCT CCA CCC TAT GAC AA* | *AAG CAA ACT TTC TGG TAG GCT T* |

**Supplemental Table 2: Patient´s characteristics.** Data are expressed as case numbers or median ± standard deviation. Abbreviations in order of their appearance: SD – standard deviation, f – female, m – male, na – not applicable, ALD – alcoholic liver disease, NAFLD – non-alcoholic liver disease, HBV – Hepatitis B virus infection, HCV – Hepatitis C virus infection, HDV – Hepatitis D virus infection, PBC – primary biliary cholangitis, PSC – primary sclerosing cholangitis, SSC – secondary sclerosing cholangitis, AIH – autoimmune hepatitis, A1-AT – alpha-1-antitrypsin deficiency associated liver disease

|  |  |  |  | Child-Pugh-Score | | | |
| --- | --- | --- | --- | --- | --- | --- | --- |
|  | N | Gender (f/m) | Age  (median+/-SD) | Healthy | A | B | C |
| Healthy volunteers | 22 | 8/14 | 62 (±13.4) | 22 | nA | nA | nA |
| Total patients | 286 | 84/202 | 63 (±11.6) | na | 151 | 71 | 64 |
| ALD | 143 | 24/119 | 64 (±9.0) | na | 59 | 43 | 41 |
| NAFLD | 70 | 31/39 | 69 (±8.5) | na | 53 | 8 | 9 |
| HBV | 9 | 3/6 | 59 (±4.6) | na | 4 | 3 | 2 |
| HCV | 38 | 9/29 | 59 (±10.1) | na | 18 | 10 | 10 |
| HDV | 2 | 1/1 | 42 (±5.5) | na | 1 | 0 | 1 |
| PBC | 11 | 10/1 | 59 (±6.0) | na | 9 | 1 | 1 |
| PSC | 1 | 0/1 | 34 (±0.0) | na | 1 | 0 | 0 |
| SSC | 2 | 0/2 | 37.5 (±15.5) | na | 2 | 0 | 0 |
| AIH | 5 | 5/0 | 51 (±7.2) | na | 2 | 3 | 0 |
| Wilson´s disease | 2 | 1/1 | 25 (±3.0) | na | 1 | 1 | 0 |
| A1-AT | 2 | 0/2 | 38 (±8.0) | na | 0 | 2 | 0 |
| Hemochromatosis | 1 | 0/1 | 65 (±0.0) | na | 1 | 0 | 0 |
