# Supplementary figures and images for: Modulation of Liver Inflammation and Fibrosis by Interleukin-37
Source: Front Immunol. 2021 Mar 4;12:603649. doi: 10.3389/fimmu.2021.603649 (PMC7970756; doi:10.3389/fimmu.2021.603649)

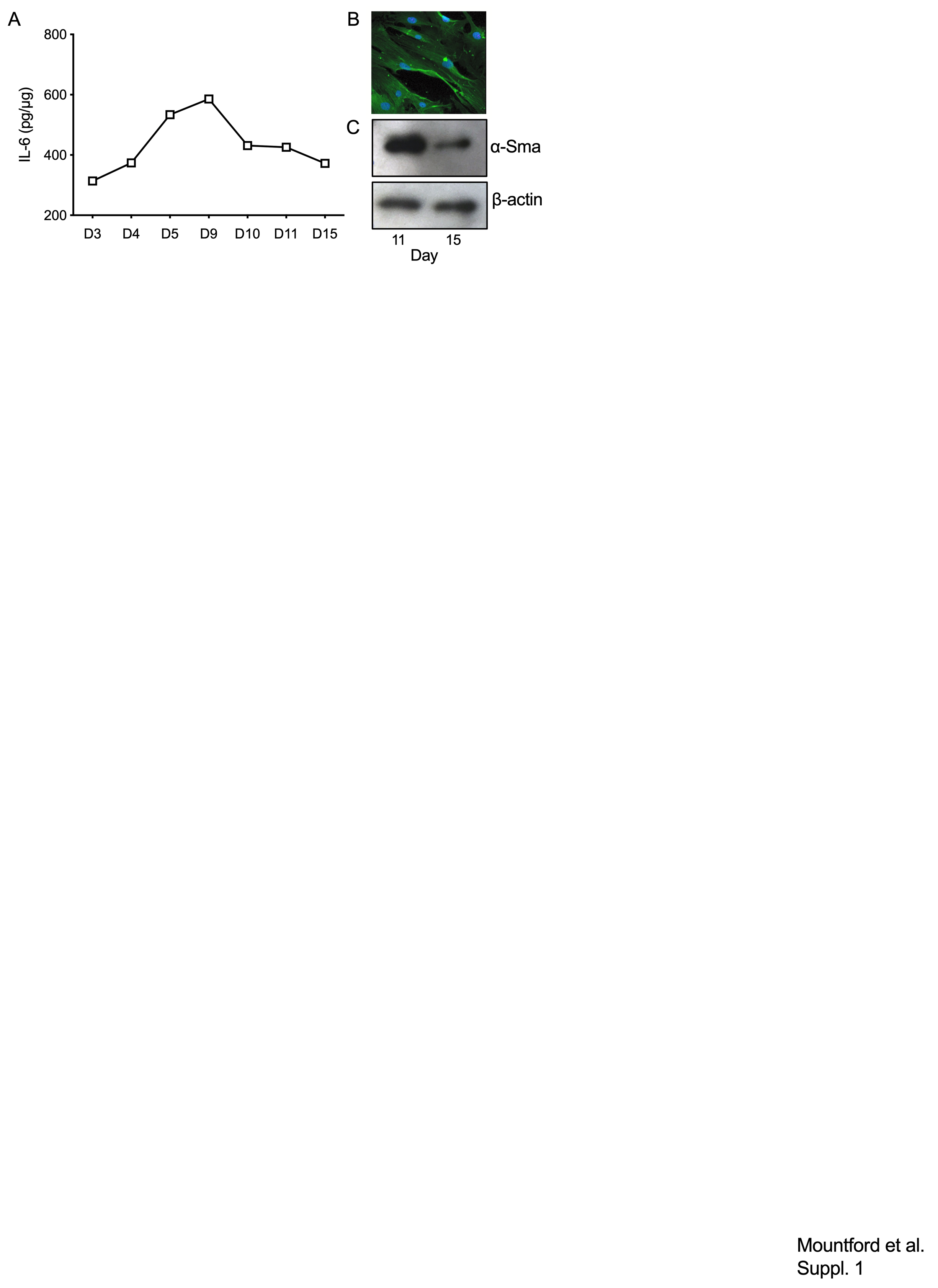

Supplement: Supplementary file 3 [file Image_1.TIFF]

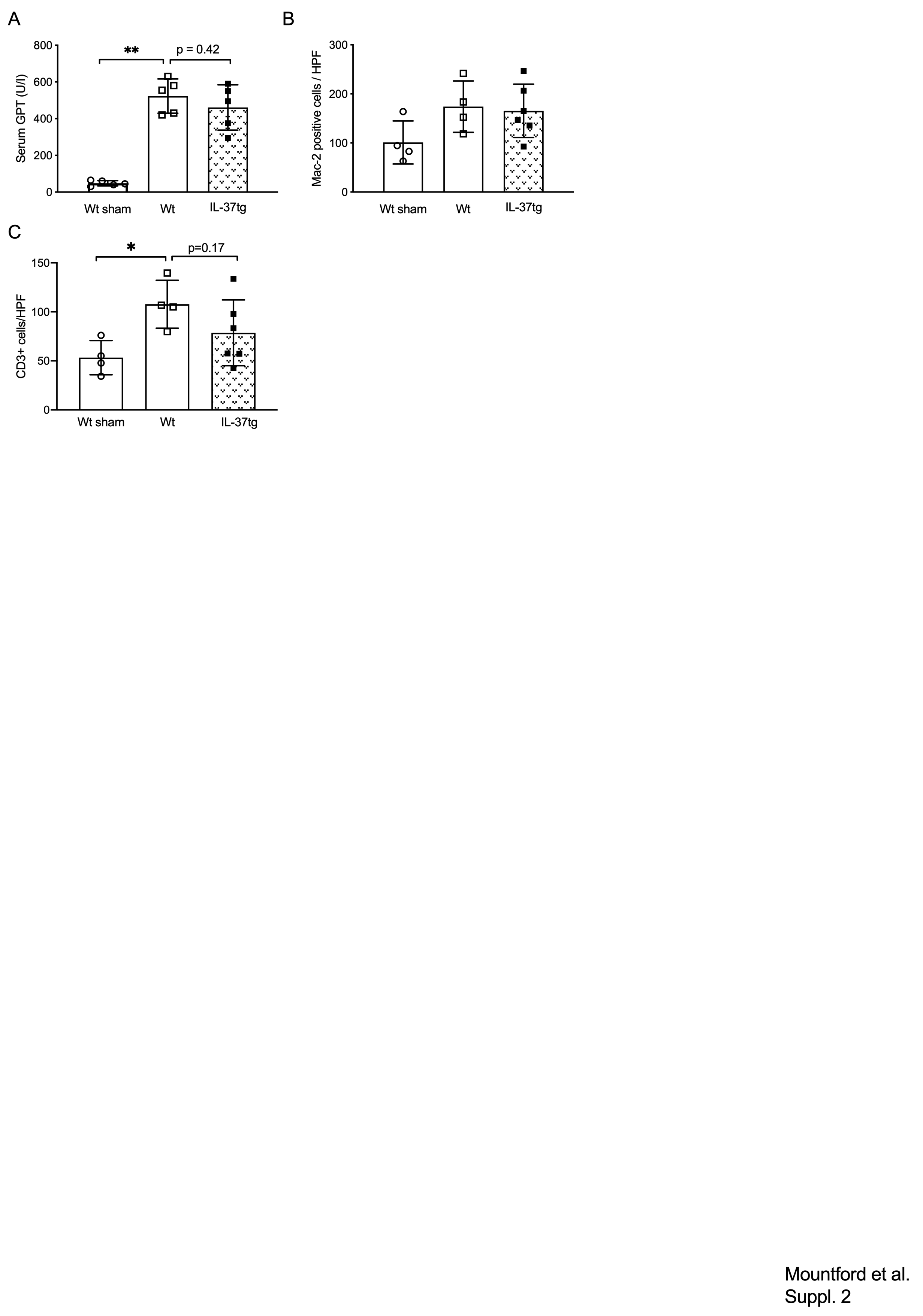

Supplement: Supplementary file 4 [file Image_2.TIFF]

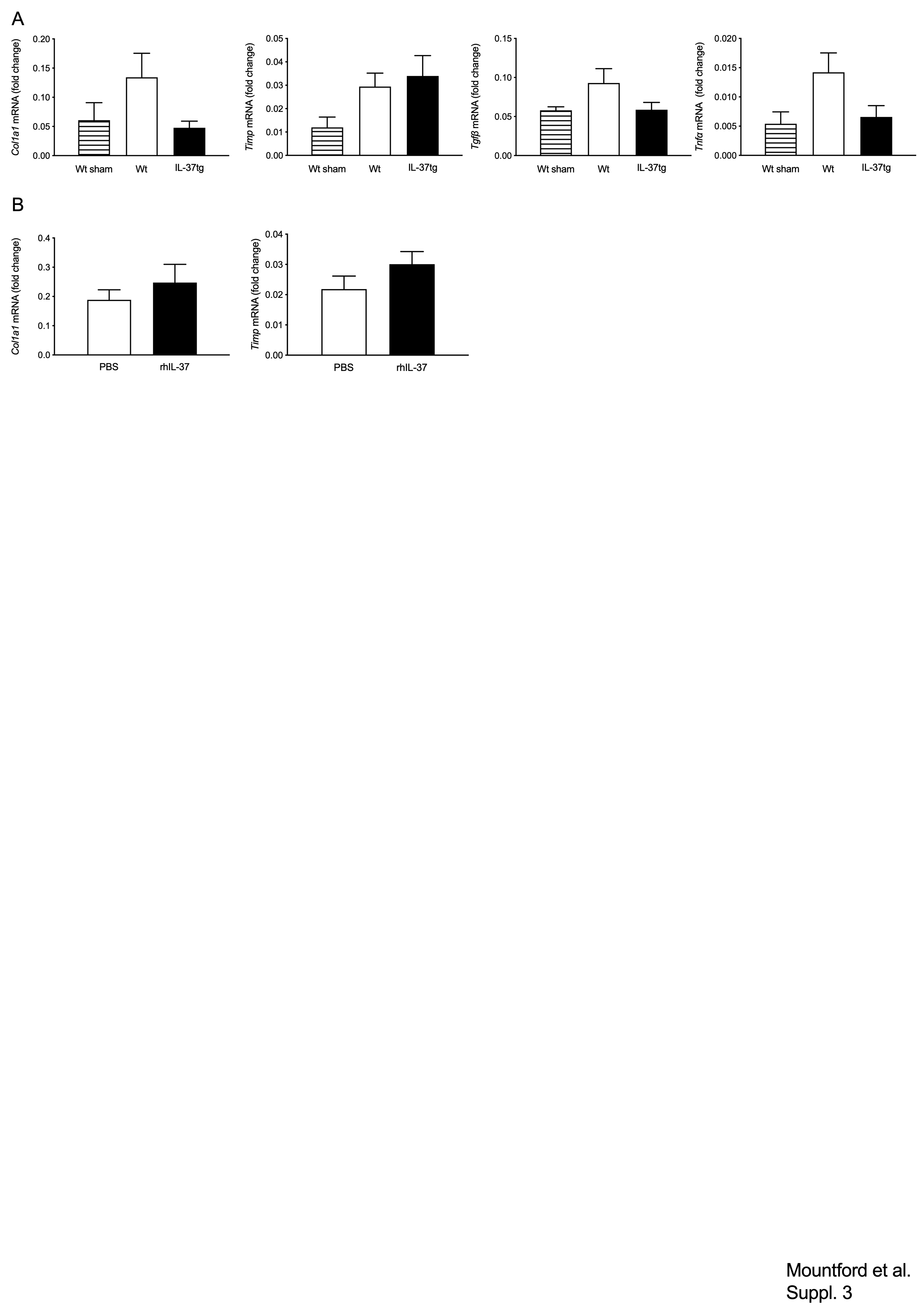

Supplement: Supplementary file 5 [file Image_3.TIFF]
